# A novel framework for the automated characterization of Gram-stained blood culture slides using a large-scale vision transformer

**DOI:** 10.1128/jcm.01514-24

**Published:** 2025-02-24

**Authors:** Jack McMahon, Naofumi Tomita, Elizabeth S. Tatishev, Adrienne A. Workman, Cristina R. Costales, Niaz Banaei, Isabella W. Martin, Saeed Hassanpour

**Affiliations:** 1 Department of Computer Science, Dartmouth College 3728 , Hanover, New Hampshire, USA; 2 Department of Biomedical Data Science, Geisel School of Medicine at Dartmouth, Hanover, New Hampshire, USA; 3 Department of Pathology and Laboratory Medicine, Dartmouth-Hitchcock Medical Center 22916 , Lebanon, New Hampshire, USA; 4 Department of Pathology, Stanford University School of Medicine 10624 , Stanford, California, USA; 5 Department of Medicine, Division of Infectious Diseases and Geographic Medicine, Stanford University School of Medicine 10624 , Stanford, California, USA; 6 Clinical Microbiology Laboratory, Stanford Health Care 474436 , Palo Alto, California, USA; 7 Department of Epidemiology, Geisel School of Medicine at Dartmouth, Hanover, New Hampshire, USA; Children's Hospital Los Angeles, Los Angeles, California, USA

**Keywords:** Gram stain, whole-slide bacterial classification, vision transformer

## Abstract

This study introduces a new framework for the artificial intelligence-based characterization of Gram-stained whole-slide images (WSIs). As a test for the diagnosis of bloodstream infections, Gram stains provide critical early data to inform patient treatment in conjunction with data from rapid molecular tests. In this work, we developed a novel transformer-based model for Gram-stained WSI classification, which is more scalable to large data sets than previous convolutional neural network-based methods as it does not require patch-level manual annotations. We also introduce a large Gram stain data set from Dartmouth-Hitchcock Medical Center (Lebanon, New Hampshire, USA) to evaluate our model, exploring the classification of five major categories of Gram-stained WSIs: gram-positive cocci in clusters, gram-positive cocci in pairs/chains, gram-positive rods, gram-negative rods, and slides with no bacteria. Our model achieves a classification accuracy of 0.858 (95% CI: 0.805, 0.905) and an area under the receiver operating characteristic curve (AUC) of 0.952 (95% CI: 0.922, 0.976) using fivefold nested cross-validation on our 475-slide data set, demonstrating the potential of large-scale transformer models for Gram stain classification. Results were measured against the final clinical laboratory Gram stain report after growth of organism in culture. We further demonstrate the generalizability of our trained model by applying it without additional fine-tuning on a second 27-slide external data set from Stanford Health (Palo Alto, California, USA) where it achieves a binary classification accuracy of 0.926 (95% CI: 0.885, 0.960) and an AUC of 0.8651 (95% CI: 0.6337, 0.9917) while distinguishing gram-positive from gram-negative bacteria.

**IMPORTANCE:**

This study introduces a scalable transformer-based deep learning model for automating Gram-stained whole-slide image classification. It surpasses previous methods by eliminating the need for manual annotations and demonstrates high accuracy and generalizability across multiple data sets, enhancing the speed and reliability of Gram stain analysis.

## INTRODUCTION

A Gram stain is a routine diagnostic test performed to help preliminarily identify the causative agent of infection. This study specifically focuses on the diagnosis of bloodstream infections (BSI), a type of infection caused by the presence of bacteria in a patient’s blood which can lead to sepsis and be life-threatening. Previous studies report that quick identification of pathogens involved in a BSI can be critical to the success of patient treatment (1). BSIs can have in-hospital mortality rates above 20%, and the correct choice of antimicrobial agent is key for the treatment of patients with BSI (2). In recent years, rapid molecular diagnostics assays, such as the Blood Culture Identification 2 (BioMérieux, Marcy l’Etoile, France), the cobas ePlex Blood Culture Identification Panel (Roche, Basel, Switzerland), and Verigene (DiaSorin, Saluggia, Italy) assays, have advanced the early detection of causative pathogens in BSIs, providing species-level identification as well as detecting key markers of antimicrobial resistance in samples from positive blood cultures (3). However, these assays must be paired with Gram stain results, either to determine whether to run a gram-positive or gram-negative panel or to corroborate the validity of the molecular result before reporting, depending on the assay.

Gram stain analysis remains a manual and time-consuming process whereby medical laboratory scientists analyze stained slides under a microscope to interpret the morphology of any microorganisms that may be present (4). Manual slide analysis consumes valuable time for trained medical laboratory scientists and risks error, more often in cases where visible bacteria are rare or poorly stained. Gram-stain error rates can vary significantly between laboratories, ranging from 0.4% to 2.7%, with discrepancies often involving missed organisms or organisms reported on Gram stain but not recovered in culture (5). Samples can also be falsely flagged for bacterial growth by monitoring systems in 1% to 10% of cases, leading to the preparation of slides with no bacteria that are especially time-consuming to analyze (6). An automated solution for Gram stain characterization can free up valuable time in clinical microbiology laboratories and enhance the efficient use of rapid molecular diagnostic assays by reducing labor demands and streamlining workflow. Additional benefits of rapid Gram stain characterization paired with molecular diagnostics include the ability to use a more targeted narrow-spectrum antimicrobial agent which can be less harmful to beneficial microbes in the body and may mitigate the development of antibiotic-resistant bacteria (7, 8).

**Fig 1:**
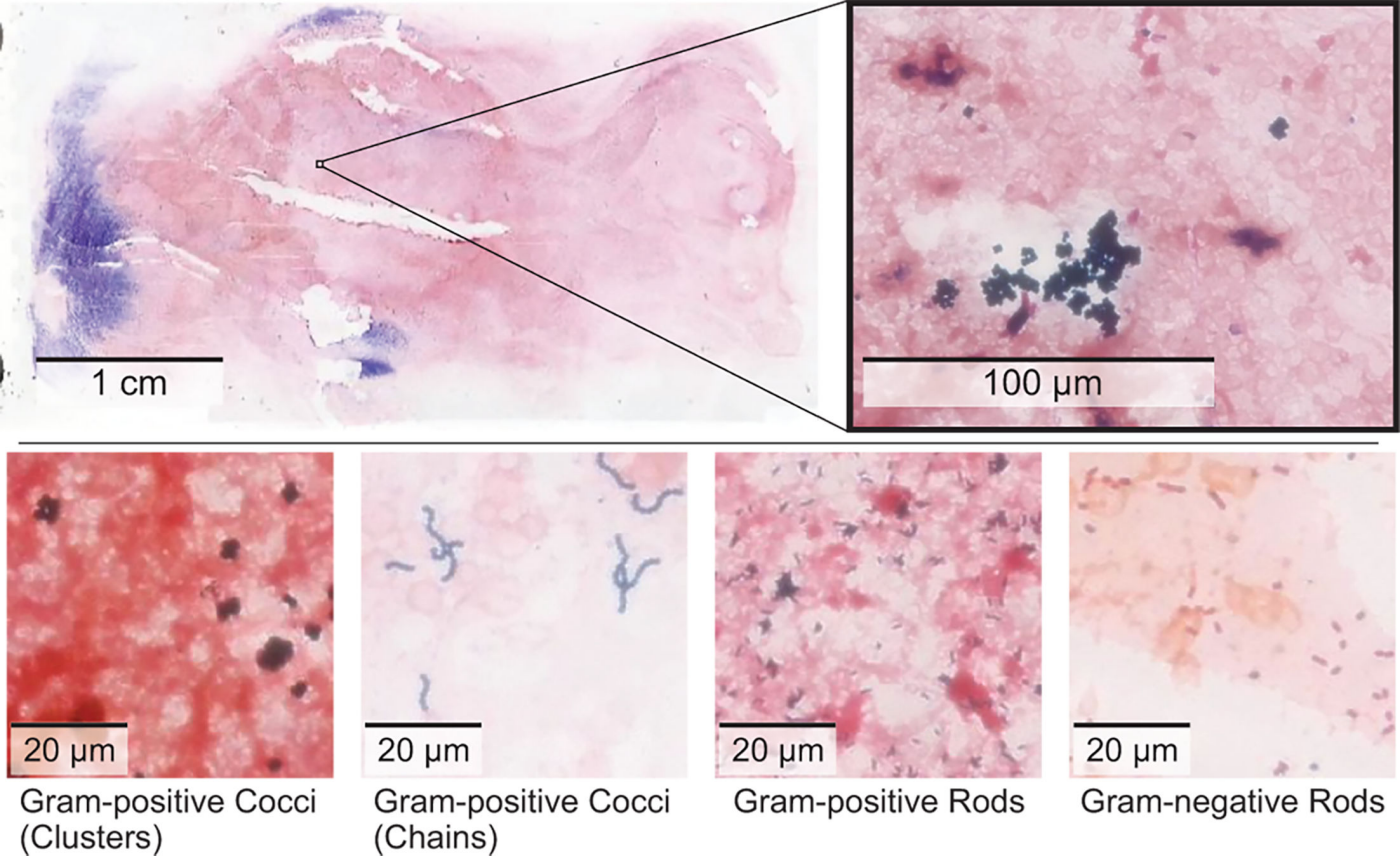
Sample images from blood cultures in the Dartmouth-Hitchcock data set, digitized at 40× magnification. Top: a scale comparison between a WSI scan and gram-positive cocci in clusters. Bottom: representative patches depicting four categories of bacterial morphologies.

Digital microscopy has been successfully combined with deep learning methods to automate slide analysis in other applications. Digital microscopy involves digitizing microscope slides into whole-slide images (WSIs), which provide a tiled view of the slide at various resolutions. WSIs typically have large file sizes, often reaching several gigabytes or more (Fig. 1). WSI analysis has been extensively explored in computational pathology, particularly for cancer diagnosis, with numerous clinical tools and specialized pipelines developed for processing WSIs as inputs to machine learning models (9, 10). Several factors have made such progress more difficult for Gram stain interpretation. Scanning microbiology slides introduces challenges that are less of an issue when scanning solid tissue, such as uneven fields of focus and background staining leading to blurry regions on WSIs (11). Residual oil on slides from clinical examination can also cause difficulties for digitization. These issues and the variable dispersion and staining of background materials and bacteria make it difficult to accurately segment Gram stain WSIs and process them in the same way as in histology slides.

Despite these challenges, previous studies have shown that deep learning methods can be effective for bacterial classification on data sets of cropped bacterial image patches (typically resized to 224 × 224 pixel dimensions or similar), distinguishing both gram-positive/negative status and clinically relevant cellular morphologies (4, 12–14). In 2018, Smith et al. introduced one of the first proof-of-concept studies for Gram-stained WSI classification. They used a convolutional neural network (CNN)-based model that was trained on manually annotated 146 × 146 pixel image patches from 189 blood culture slides and aggregated predictions from patches to obtain a WSI-level label. Using this approach, they demonstrated a classification accuracy of 92.5% while predicting slides across four separate classes, with the result excluding slides misclassified as background. This work also developed the concept of using deep learning models to extract diagnostically relevant patches for microbiologist review (11). In 2021, Alhammad et al. used manually labeled patches to train a CNN to identify and remove background areas prior to further analysis of Gram-stained WSIs, and in 2024, Walter et al. developed a CNN-based model as a clinical tool for the analysis of Gram-stained WSIs (6, 15). The model was evaluated by trained microbiologists and, rather than making a WSI-level prediction, it was designed to identify and classify diagnostically relevant image patches for review by microbiologists.

In this work, we propose GramViT, a novel vision transformer approach for automating Gram stain analysis, building upon recent advances in computer vision and digital pathology. The transformer architecture has underpinned some of the most important recent advances in deep learning, such as ChatGPT, using self-attention to model complex relationships in sequences of input data (16). Vision transformers have been used successfully to improve model performance and remove the need for manual patch-level annotations in computational pathology (17–19). Self-attention allows vision transformers to learn to identify diagnostically relevant slide regions in a self-supervised manner, enabling the creation of unprecedentedly large foundation models for computational pathology (20, 21). Recently, LongNet and LongViT have been introduced as an effective approach to train on gigapixel-sized images using dilated attention (22, 23). GramViT lays out a framework to apply these techniques to Gram stain analysis, using a pre-trained LongViT model to generate embeddings for large 4,096 × 4,096 pixel regions, which are randomly sampled during training and systemically aggregated during inference to obtain WSI-level predictions. The self-attention mechanism of the transformer architecture is able to identify the location of the bacteria in an image and then focus on those areas while making predictions for an entire slide region. This represents an improvement over previous approaches which required extensive annotations of bacteria within an image to train models using a CNN model architecture. By this approach, our work aims to bridge the gap toward developing a more robust model for Gram stain analysis that can be trained without manual patch-level annotations and scale efficiently to larger data sets.

## MATERIALS AND METHODS

### Data sets

This study introduces a newly collected Gram stain data set from Dartmouth-Hitchcock Medical Center (DHMC), a tertiary medical center in Lebanon, NH. Between August 2023 and July 2024, a total of 516 Gram-stained blood culture slides obtained during routine patient care were collected, deidentified, assigned a study number, and digitized into WSIs using a Grundium OCUS40 microscope scanner (Grundium, Tampere, Finland) at 40× magnification (0.25 µm/pixel). Slides were prepared from patient samples reported positive for potential bacterial growth by the BD BACTEC Blood Culture System (Becton, Dickinson, and Company, Franklin Lakes, NJ). The data collection and usage in our study were determined to be “Exempt” by the Dartmouth-Health Institutional Review Board. Data were collected under a waiver of informed consent. The study was designed to include the five most common slide types: (i) gram-positive cocci (GPC) in clusters, (ii) GPC in pairs/chains, (iii) gram-positive rods (GPRs), (iv) gram-negative rods (GNRs), and (v) No bacteria. Other categories, such as gram-negative cocci, yeast, or slides with multiple morphologies, were not included due to the rarity of these types in the DHMC’s cohort. After a quality assurance review, 26 scanned slides were excluded from the study as they fell into rare categories rather than the five major categories considered in this study. Additionally, 15 other scanned slides were excluded due to digitization or staining artifacts, or poor image focus quality. The remaining 475 Gram-stained slides were included in the study, as shown in Table 1. Of the 475 included slides, 11 were initially reported incorrectly by laboratory technologists and later corrected, representing an error rate of 2.32%. The reference standard for slides included in this study was established based on the final clinical laboratory report to include review of initial Gram stain in cases of discrepancy between Gram stain and culture growth.

**TABLE 1:** Statistics of the DHMC data set: WSI counts across selected bacterial subgroups

Bacterial subgroup	WSI count
Gram-positive cocci in clusters	184
Gram-positive cocci in pairs/chains	68
Gram-positive rods	37
Gram-negative rods	122
No bacteria	64
Total	475

To demonstrate the generalizability of our approach, we utilized two external data sets for additional validation (Table 2). The first external data set consists of medium-sized cropped Gram stain images provided by collaborators at Stanford Health. This data set contains one to three large images per slide, scanned at 80× magnification using MoticEasyScan Infinity Scanner (Motic, Hong Kong), collected from a total of 32 slides. These include blood culture infections and samples from various other infection sites, such as wounds and cerebrospinal fluid. Due to the limited data set size and the lack of detailed labels for many slides, we focused on binary classification between gram-positive and gram-negative bacteria. After excluding slides with multiple or no bacterial labels, 27 slides remained in the Stanford data set. The second external data set is a publicly available test set comprising 1,000 small image crops collected at Medical Faculty Mannheim, Heidelberg University (MHU), from sepsis patients between 2015 and 2019 (9, 24). The MHU data set contains images classified as either gram-positive or gram-negative; however, details regarding the distribution of images per slide, the scan resolution, and the scanner model were not provided.

**TABLE 2:** Statistics of the external data sets: Stanford and MHU data sets^*a*^

Data set	Classification scope	Labels	Test set
Stanford	Whole-slide level	Gram-positive/gram-negative	27 Slides
MHU	Patch level	Gram-positive/gram-negative	1,000 Images

^*a*^ The Stanford data set was used to assess WSI-level predictions, while the MHU data set was used to evaluate patch-level predictions.

### Data preprocessing

**Fig 2:**
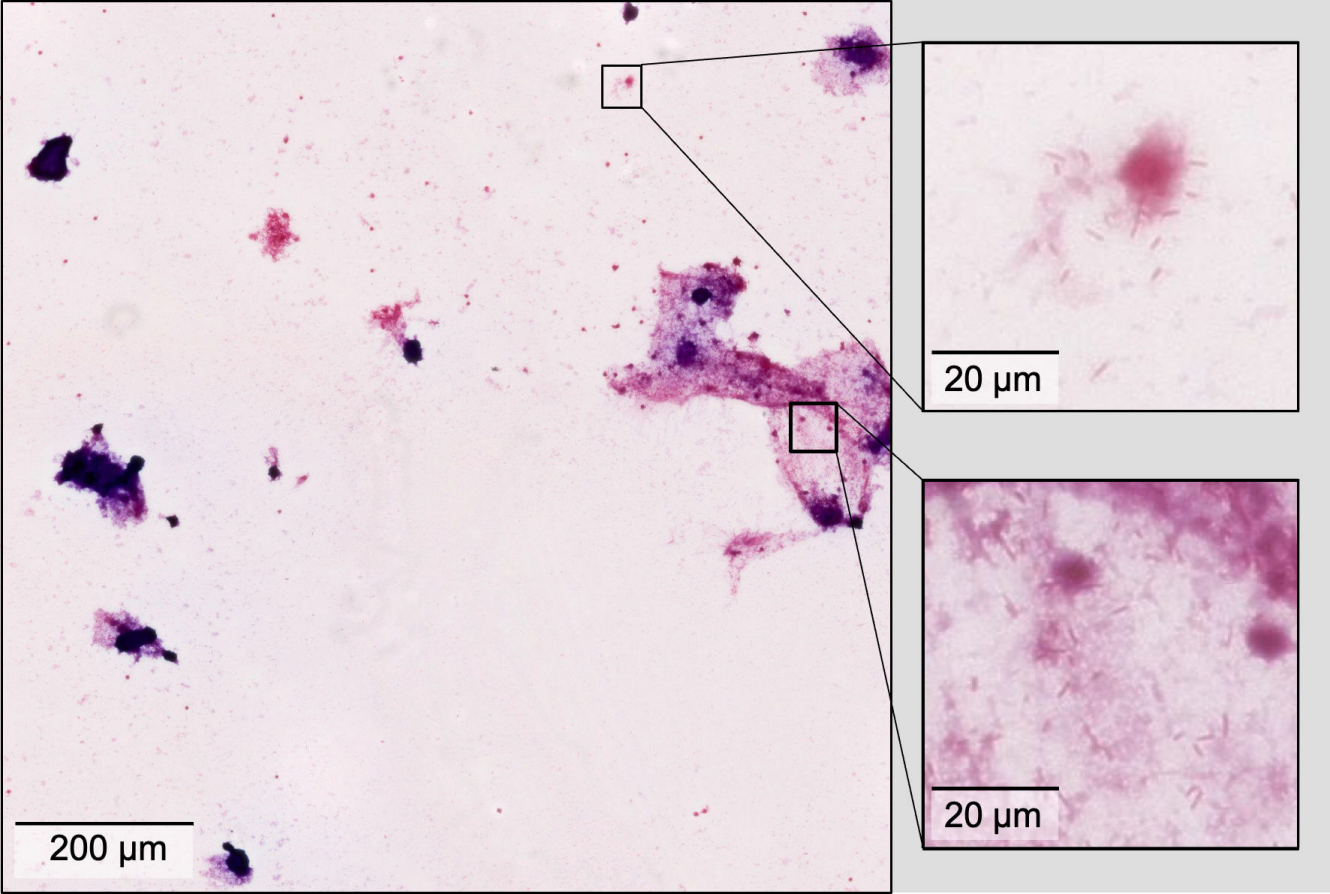
A representative 4,096 × 4,096 region at 40× magnification extracted from a slide with gram-negative rods in the DHMC data set. Inset windows show bacterial size and distribution. Unlike previous methods with smaller training regions, GramViT trains on large regions that capture diverse bacterial distributions and background material, improving region-level predictions. Larger region size also increases the likelihood of matching region-level labels to slide-level labels, crucial for effective model training.

The OpenSlide library was used for reading and processing the WSIs stored in SVS file formats. A simple image thresholding algorithm was first applied to slides at a lower resolution to identify regions with a color profile consistent with stained material. A sliding window approach was then used to extract non-overlapping 4,096 × 4,096 pixel regions that contained at least 15% stained material. Optical focus quality can be a significant issue with Gram-stained slides. To address this, regions were further filtered to exclude those with low Laplacian variance, a method that can flag blurry images (24). The number of regions extracted can vary widely depending on the slide, as some slides were almost entirely covered with sample material, while others had large blank spaces. For about 90% of slides, 30 to 750 regions were extracted, with a median of 347. In total, 177,485 regions were extracted from the entire 475-slide data set (Fig. 2).

### GramViT training pipeline

Our pipeline uses the LongViT vision transformer architecture, which has been pre-trained using The Cancer Genome Atlas data set to extract histopathological features and generate embeddings for gigapixel-sized histology images (23). Vision transformers, such as LongViT, require large amounts of training data to accurately learn the complex relationships necessary for extracting diagnostically relevant information from whole-slide images. Due to this requirement, it is common to first pre-train a model using self-supervised learning on large data sets to learn how to generate a meaningful embedding. LongViT was pre-trained using the self-distillation with no labels (DINO) framework for this stage (25). Fine-tuning a pre-trained LongViT model, rather than starting from scratch, allows us to mitigate the challenge posed by the relatively limited size of the DHMC Gram stain data set. This approach aligns with the transfer learning paradigm, commonly used in other areas of medical image analysis, where pre-trained models are adapted for tasks with limited data availability (26).

**Fig 3:**
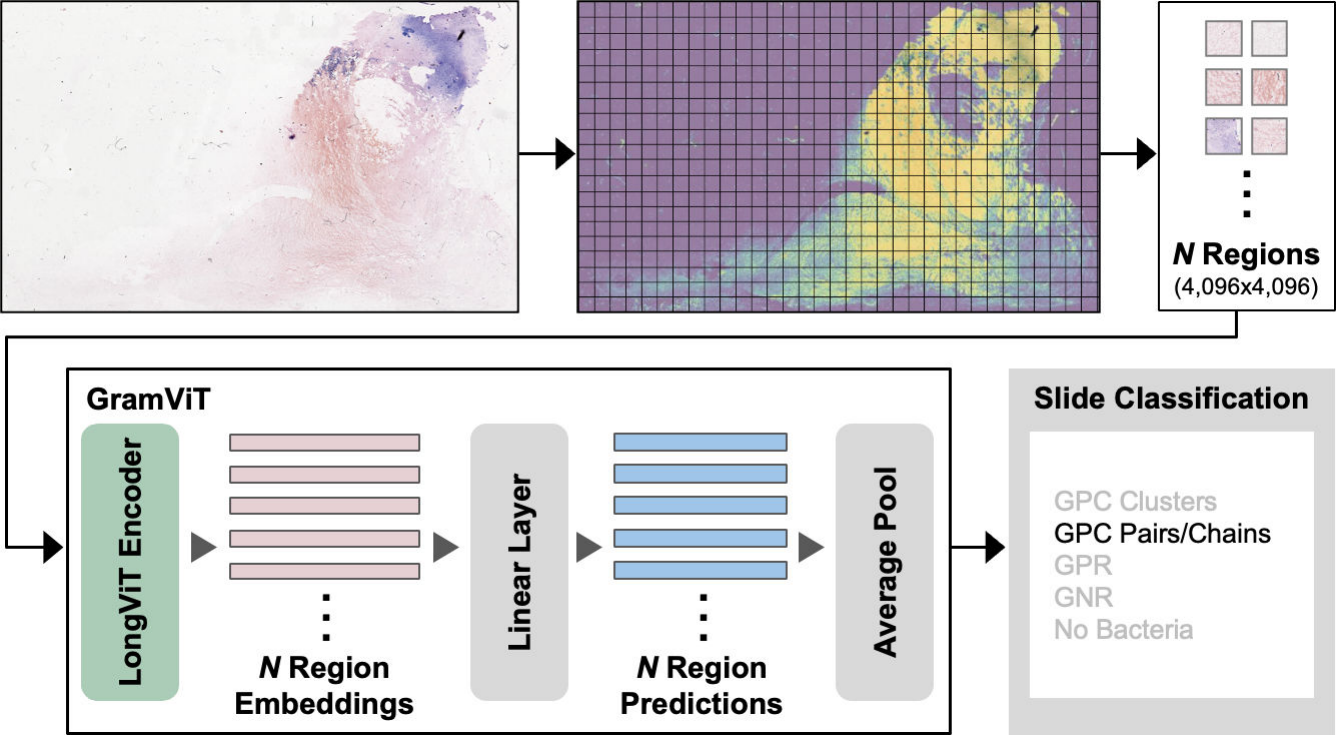
Overview of the GramViT training process: A Gram-stained WSI was divided into a grid of non-overlapping regions, with background regions identified and filtered out. A specific number of regions were selected from each sampled WSI to train the LongViT encoder, which produced a 384-dimensional embedding per region. A linear classification layer was then applied to predict class outputs for each embedding, and average pooling of regional class predictions was used to generate a WSI-level classification. During testing, our approach systematically sampled every region of a given WSI for the final inference.

Our training process involved replacing the model’s final layer with a linear classification layer that maps the 384-dimensional embedding output of LongViT to the five output classes in this study. During each training epoch, sampling a WSI involved extracting a single 4,096 × 4,096 pixel region to fine-tune the model (Fig. 3). We sample each WSI multiple times per epoch and apply oversampling to minority classes to account for class imbalances during training. Training loss is calculated using cross entropy.

During the testing phase, each region is systematically sampled, and class predictions are average-pooled across regions before calculating performance metrics at the WSI prediction level. GramViT is optimized through our region-sampling approach during training. Relatively large image regions are used as model inputs, rather than downscaling the images during fine-tuning, unlike the approach in the original LongViT paper (23). This is because, in Gram stain analysis, the model relies more on preserving finer details of individual cells rather than capturing larger structures, as is common in cancer histology slide analysis. In our case, downscaling can obscure the distinctions between similar morphologies, such as gram-positive rods and gram-positive cocci in pairs/chains. This effect is explored in an ablation study on input image resolution, which is included in the Results section. Additionally, we have access to a relatively small data set of 475 WSIs in this study. Therefore, using a random region-sampling approach in our training process can be beneficial for increasing the effective training data. Unlike cancer histology slides, Gram-stained blood culture slides originate from a liquid medium, which tends to distribute diagnostically relevant regions more uniformly. As a result, bacteria are typically smeared consistently across visibly stained regions of the slide, and a sufficiently large region chosen at random is likely to contain bacteria corresponding to the slide-level label.

### Evaluation metrics and statistical analysis

Model predictions are compared to the ground truths for each slide, as listed in the clinical laboratory report. We report model performance using F1 score, accuracy, and area under the receiver operating characteristic curve (AUC), as well as precision, recall, sensitivity, and specificity on a per-class basis. Average metrics are reported using micro-averaging to account for class imbalances and to better reflect performance across the typical composition of slides encountered by microbiology laboratories. The exception is AUC, which is reported using macro-averaging. All metric implementations are sourced from the scikit-learn library (Scikit-learn Consortium, Inria Foundation), and 95% confidence intervals (95% CIs) are computed using bootstrapping.

## RESULTS

Experiments were conducted using fivefold nested cross-validation on the DHMC Gram stain data set. In each iteration of fivefold cross-validation, 60% of the data were used for training, with 20% used for validation and 20% used for testing. As discussed above, due to the limited distribution of certain types of bacterial morphologies in the data set, experiments focused on classification between the five most common Gram-stained slide types (gram-positive cocci in clusters, gram-positive cocci in pairs/chains, gram-positive rods, gram-negative rods, and no bacteria). Slides that contained yeast, gram-negative cocci, or multiple labels were excluded from our study.

**Fig 4:**
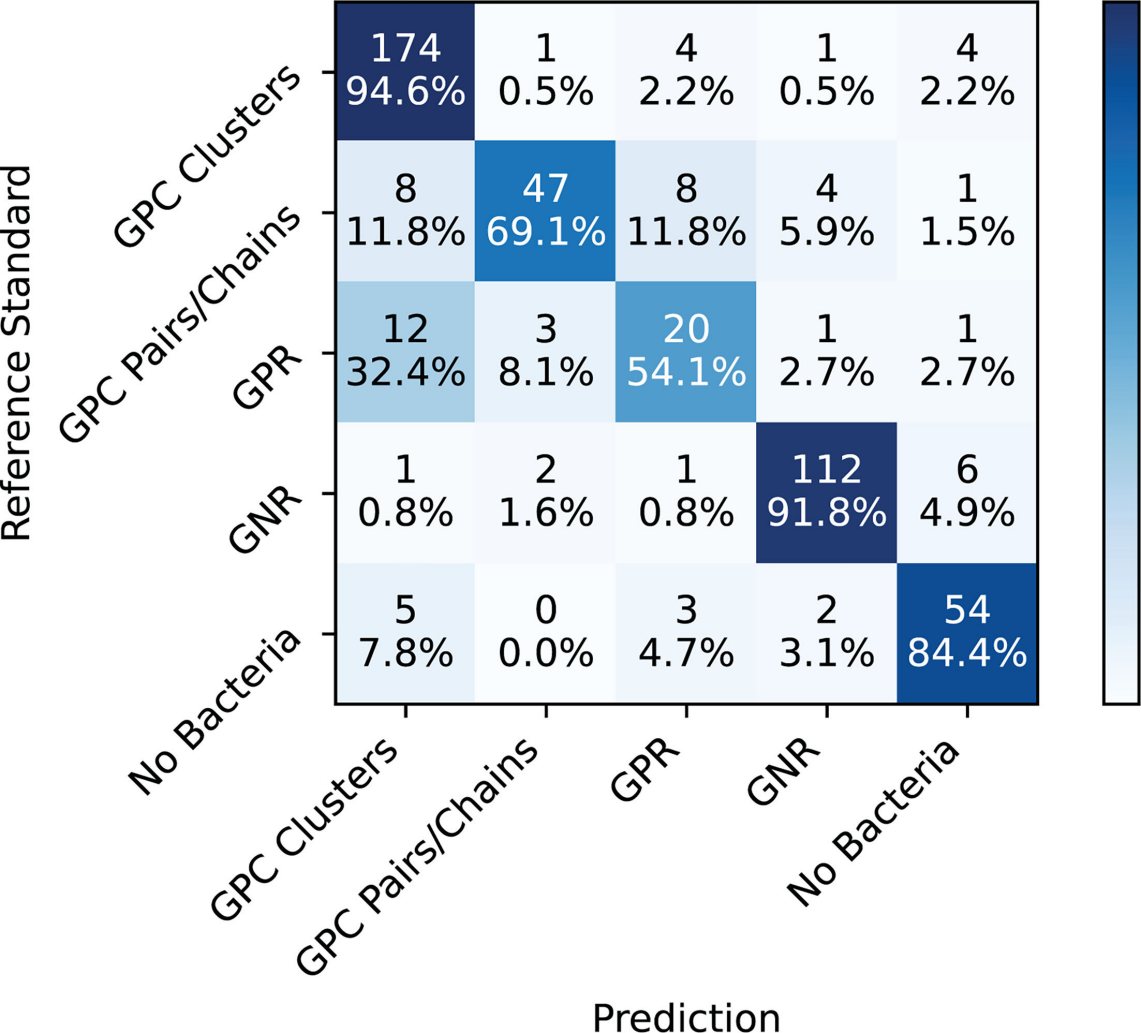
GramViT Confusion Matrix with precision/recall breakdown across five bacterial morphologies. Comparing model predictions on the DHMC test set using fivefold nested cross-validation to ground truths determined by the final clinical laboratory Gram stain report after growth of organism in culture.

**Fig 5:**
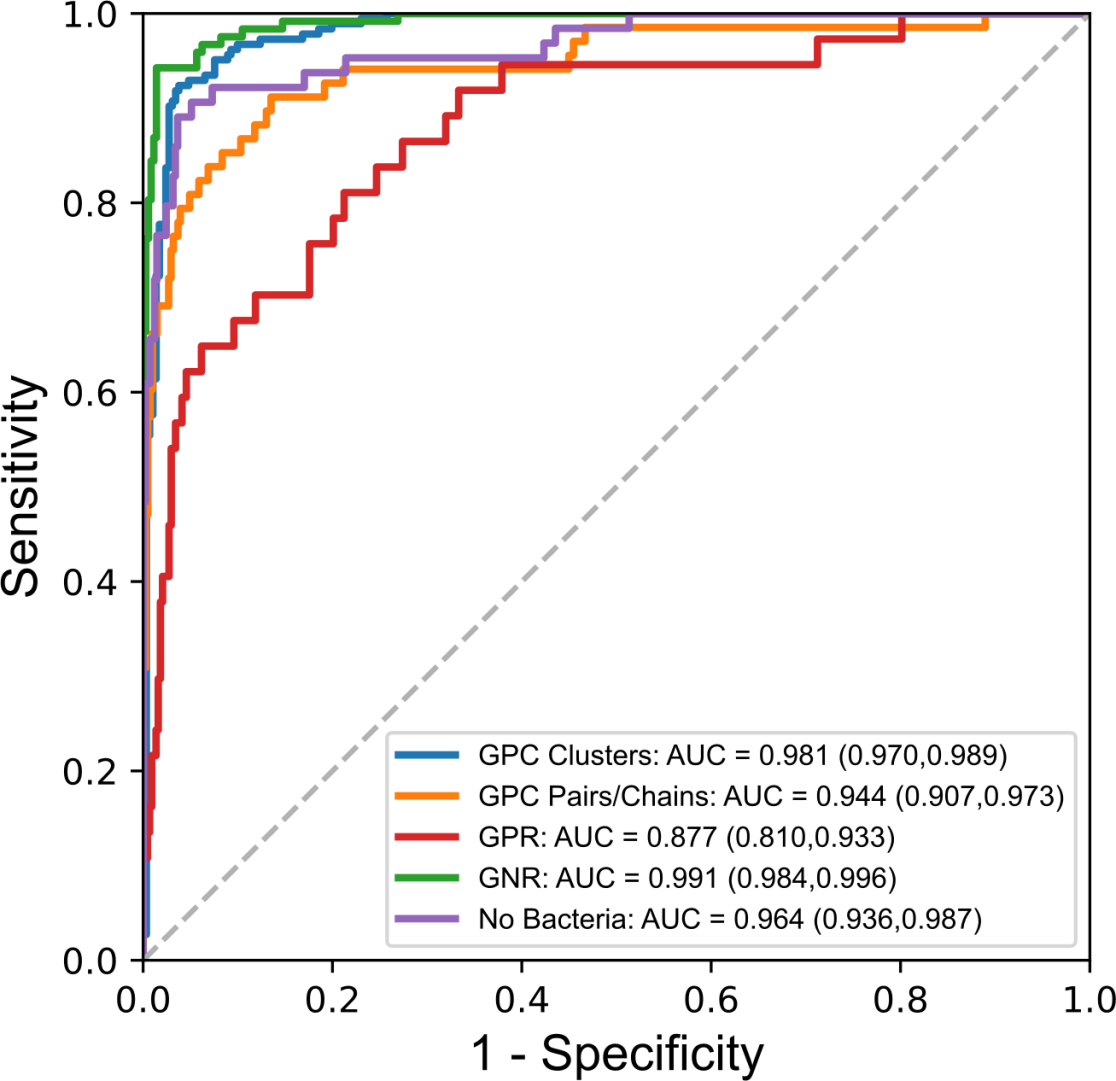
GramViT receiver operating characteristic curves and associated area under the curve compare the model performance across each of the five classes of WSIs in the DHMC test set.

The experimental setup used 40× magnification and a region size of 4,096 × 4,096 pixels. Regions were sampled from a grid with no overlap, with one region sampled per WSI during model training. Every region in a WSI was sampled systematically during validation and testing to obtain a stable and comprehensive prediction for each slide. Training used a learning rate of 5e-5 and five warmup epochs. We also used a batch size of 8 and an update frequency of every three steps. Models were trained for 30 epochs, with each WSI sampled up to 11 times per epoch depending on class labels to account for class imbalances. Each fold was trained using a Nvidia RTX A6000 GPU (Nvidia, Santa Clara, CA). Testing results aggregate performance on the test set across each of the five folds (Fig. 4 and 5).

Overall, GramViT achieved an accuracy of 0.857 (95% CI: 0.810, 0.900) and an AUC of 0.952 (95% CI: 0.922, 0.976). When considering the breakdown of results by category, there is a strong gap in performance between well-represented slide types (i.e., GPC in clusters and GNR), less well-represented slide types (i.e., GPC in pairs/chain and no bacteria), and poorly represented slide types (i.e., GPR) in the data set distribution (Table 3). Of note, among the 11 slides initially misclassified by laboratory technologists, GramViT correctly predicted the bacterial class in 8.

**TABLE 3:** Results of GramViT predictions by class on the DHMC Gram stain data set using region resolution at 40× magnification, with 95% confidence intervals^*a*^

Morphology	F1 score	Precision	Recall/Sensitivity	Specificity
GPC in clusters	0.906 (0.855, 0.948)	0.871 (0.800, 0.938)	0.945 (0.892, 0.988)	0.910 (0.862, 0.958)
GPC in pairs/chains	0.774 (0.619, 0.880)	0.888 (0.731, 1.000)	0.692 (0.500, 0.842)	0.985 (0.965, 1.000)
GPR	0.539 (0.273, 0.739)	0.555 (0.273, 0.812)	0.540 (0.278, 0.778)	0.964 (0.936, 0.989)
GNR	0.925 (0.868, 0.971)	0.934 (0.867, 1.000)	0.917 (0.833, 0.981)	0.978 (0.953, 1.000)
No bacteria	0.827 (0.704, 0.926)	0.816 (0.655, 0.950)	0.843 (0.696, 0.964)	0.970 (0.942, 0.994)
*Micro-average^b^*	*0.857 (0.810, 0.900*)	*0.857 (0.810, 0.900*)	*0.857 (0.810, 0.900*)	*0.965 (0.951, 0.976*)

^*a*^ The micro-average represents the average performance across all instances, where each instance is equally weighted, regardless of class distribution.

^*b*^ Italic font emphasizes the distinction of all reported numbers.

To benchmark results for GramViT against a CNN-based model, we used the Deepslide framework (10). Deepslide is a sliding window based framework for microscopy image classification that we trained on the DHMC data set using patches of size 224 × 224 pixels without patch-level annotations. The same stain-filtering extraction methodology and fivefold nested cross-validation evaluation were used for both models. We observed that GramViT had higher accuracy, F1, and AUC scores than Deepslide in all metrics (Table 4).

**TABLE 4:** GramViT prediction results benchmarked against a CNN-based model using the Deepslide framework, with 95% confidence intervals

Model	Accuracy	F1 score	AUC
Deepslide	0.771 (0.710, 0.820)	0.771 (0.705, 0.830)	0.908 (0.880, 0.930)
GramViT	0.857 (0.810, 0.900)	0.857 (0.810, 0.900)	0.952 (0.922, 0.976)

### Ablation study

We conducted an ablation study to validate our choice of region dimensions and image resolution for model training. This study evaluates the tradeoff between longer sample processing duration and improved model performance, helping to determine the optimal configuration for our approach. In total, we trained four models on either 4,096 × 4,096 or 1,024 × 1,024 pixel input regions, at 40× magnification or down-sampled to 20× magnification. To balance the amount of training data available to each model per epoch, the 1,024 × 1,024 pixel models were trained on 16 regions per sampled WSI while the 4,096 × 4,096 pixel models were trained on only one region per sampled WSI. This evaluation is especially important for Gram stain analysis because it investigates our hypothesis that transformer models will become more effective at bacteria classification and background discrimination when trained on larger input regions. Investigating the effect of different input resolutions aims to determine whether down sampling, a technique that could lead to faster processing and training times, negatively impacts model performance. Our results suggest that down sampling to 20× magnification could, in fact, reduce model performance, though this negative impact is less than that of input region size (Table 5).

**TABLE 5:** Ablation study comparing the impact of input region size and resolution on GramViT model performance^*a*^

Model input	Accuracy	F1 score	AUC
1,024 × 1,024 at 20×	0.756 (0.717, 0.794)	0.756 (0.717, 0.794)	0.899 (0.871, 0.921)
1,024 × 1,024 at 40×	0.787 (0.751, 0.824)	0.787 (0.751, 0.824)	0.916 (0.891, 0.937)
4,096 × 4,096 at 20×	0.793 (0.757, 0.83)	0.793 (0.757, 0.83)	0.927 (0.905, 0.945)
4,096 × 4,096 at 40×	0.865 (0.835, 0.896)	0.865 (0.835, 0.896)	0.964 (0.948, 0.976)

^*a*^ Models were trained for 30 epochs using five-fold cross-validation and evaluated for performance on the validation sets, totaling 475 slides across all folds.

### Evaluation on external data set

To ensure that GramViT is robust in the presence of variability in bacterial morphology and staining techniques and generalizes beyond the DHMC data set, we validated the trained model on two external data sets (Table 2). Extracted regions from the Stanford data set were down-sampled to 40× resolution. In cases where the Stanford data set contained multiple crops from a single slide, we aggregated regions across all crops to make a single slide-level prediction in a manner consistent with that used on the DHMC data set. Since the MHU data set contains small image crops and does not include slide-level information, each crop was characterized independently. This is consistent with the approach used in the original MHU study. For both data sets, GramViT was trained on the DHMC data set and then applied directly to characterize slides without any fine-tuning (Table 6).

**TABLE 6:** Results compare models trained on the DHMC data set and applied for binary classification between gram-positive and gram-negative bacteria on each external data set

Data set - Method	Accuracy	F1 score	AUC
Stanford - Deepslide	0.702 (0.640, 0.760)	0.702 (0.640, 0.760)	0.675 (0.366, 0.963)
Stanford - GramViT	0.926 (0.885, 0.960)	0.926 (0.885, 0.960)	0.865 (0.634, 0.992)
MHU - PoolFormer (27)	0.951^*a*^	–	–
MHU - Deepslide	0.528 (0.455, 0.600)	0.528 (0.455, 0.600)	0.528 (0.505, 0.552)
MHU - GramViT	0.898 (0.855, 0.935)	0.898 (0.855, 0.935)	0.951 (0.948, 0.954)

^*a*^ The PoolFormer model was trained on the MHU data set, and its result is sourced from the original MHU study. F1 score and AUC were not reported for this model.

We also compared the performance of GramViT with a CNN-based Deepslide model for external data set evaluation. Both models were trained on the DHMC and evaluated for binary classification on external data sets without fine-tuning. GramViT demonstrated significantly better generalization on the Stanford data set, achieving an AUC of 0.8651 (95% CI: 0.6337, 0.9917), compared to Deepslide’s AUC of 0.675 (95% CI: 0.366, 0.963). A similar trend was observed with the MHU data set, where GramViT achieved an AUC of 0.9507 (95% CI: 0.9477, 0.9539) versus Deepslide’s AUC of 0.528 (95% CI: 0.505, 0.552). However, neither model matched the accuracy reported with the PoolFormer model in the original paper (27).

## DISCUSSION

This study proposes GramViT, a framework that applies vision transformer-based methodologies to the classification of Gram-stained WSIs. GramViT provides a weakly supervised training solution for Gram-stained WSIs, addressing the bottleneck of manual patch-level data annotation required for scaling model training on larger data sets. Our results, an accuracy of 0.857 (95% CI: 0.810, 0.900) and an AUC of 0.952 (95% CI: 0.922, 0.976), demonstrate the model’s ability to accurately characterize and classify Gram-stained WSIs. Notably, GramViT successfully identified 8 out of the 11 slides from the DHMC data set that had been misclassified in initial laboratory reports, highlighting its potential to catch details that may be missed by laboratory technologists when analyzing slides.

This work advances prior methods by incorporating significantly larger input regions for weakly supervised model training. Unlike CNN-based models, which typically rely on small 224 × 224 pixel input patches, GramViT processes 4,096 × 4,096 pixel regions, large enough to reliably encompass bacteria given their typical dispersion across smeared slides. We leverage a LongViT model pre-trained for general WSI interpretation across diverse histopathology data sets, enabling the model to learn broad patterns in whole-slide images. Unlike CNN-based models that require manual annotations to specify the location of each bacterium, the ViT automatically learns regions of focus by analyzing the entire image using a slide-level label as the sole guidance signal. By learning patterns from the data, it identifies essential features, such as bacteria, and their locations, without requiring explicit human input. Subsequently, we fine-tune the model’s embeddings to capture Gram stain-specific features, enhancing its capability to analyze Gram-stained slides.

The benefits of GramViT’s large input regions over CNN-based models are evident in the head-to-head comparison presented in this work. GramViT achieved superior characterization of slides within the DHMC data set, with an accuracy of 0.857 (95% CI: 0.810, 0.900) compared to 0.771 (95% CI: 0.710, 0.820) for the Deepslide CNN-based model. These findings support the prior comparison of transformer and CNN models for Gram stain analysis conducted by Kim et al., while extending the evaluation to include holistic methods for WSI analysis (27). However, the true strengths of our vision transformer-based approach become more evident when evaluating model performance on external data sets. A clinically useful Gram stain classification model must generalize to bacterial features across diverse laboratory and clinical settings. Scans of Gram-stained slides exhibit variability in lighting, color profile, and stain characteristics based on the scanners and staining protocols used (5). GramViT excels in binary classification without fine-tuning, achieving an accuracy of 0.926 (95% CI: 0.885, 0.960) on the Stanford data set and 0.898 (95% CI: 0.855, 0.935) on the MHU data set. In contrast, the CNN model struggles to perform, with accuracies of 0.702 (95% CI: 0.640, 0.760) and 0.528 (95% CI: 0.455, 0.600) on the Stanford and MHU data sets, respectively. This highlights the vision transformer’s ability to identify robust and transferable features essential for bacterial characterization, regardless of laboratory-specific staining and scanning variations. While publicly available data sets for Gram stain analysis remain limited, the external data sets utilized in this study provide valuable insights into GramViT’s performance. Although these data sets differ from the DHMC data set in terms of gold standard definitions and slide types (e.g., non-blood culture sources), they serve as useful benchmarks for assessing the model’s robustness and its ability to generalize across different clinical settings. Despite GramViT’s strong generalizability, validating its performance on slides prepared in different time periods is essential to ensure consistency and robustness for clinical use. Additionally, this pipeline will benefit from an AI-based pre-screening module to automatically detect and flag slides with artifacts or poor image quality, routing them for manual review to ensure accurate analysis.

While GramViT’s performance on the MHU data set is strong, with an accuracy of 0.898 (95% CI: 0.855, 0.935), the PoolFormer model reported in the original paper still outperforms it with an accuracy of 0.951. Notably, the PoolFormer model was specifically trained on the MHU training set for small-crop classification, whereas GramViT, trained on the DHMC data set for WSI-level classification, was applied out-of-the-box without prior exposure to small image crops. Although fine-tuning on the MHU data set could improve GramViT’s performance, these strong out-of-the-box results are promising. Any practical implementation of automated characterization tools for Gram stain analysis will need to perform well in new hospital settings without requiring extensive fine-tuning for each specific setup.

Furthermore, our study provides evidence that Gram stain interpretation benefits from using larger input regions at higher resolution. The ablation study demonstrates that the model trained on 4,096 × 4,096 pixel regions at 40× magnification outperforms those trained with smaller 1,024 × 1,024 pixel regions or 20× magnification. This indicates potential for further improvements through higher magnification beyond 40× magnification or the utilization of larger input regions.

One of the main limitations of this study is the gap in performance between GramViT and manual reads by laboratory technologists. The higher error rate for the GramViT analysis can be attributed to the limited size and imbalance of the training data set, which currently limits the model’s performance on more challenging cases. Future work will focus on expanding the size and diversity of the training data set to improve model accuracy and address these limitations. Common applications of transformers in computational pathology often utilize data sets containing 2,000 to 10,000 slides or more, indicating that larger data set sizes could yield substantial benefits (19, 23). This is further supported by our findings, where the model’s performance was notably better for bacterial classes that were more prevalent in the data set. Despite oversampling minority classes during training to address class imbalance, gram-positive cocci in clusters and gram-negative rods were identified far more accurately than other bacterial types. For example, gram-positive cocci in clusters, with 184 WSIs, had a sensitivity (recall) of 0.945 (95% CI: 0.892, 0.988), whereas gram-positive rods, with only 37 WSIs, had a sensitivity (recall) of 0.540 (95% CI: 0.278, 0.778). This suggests that increasing the amount of data, particularly for less common bacterial morphologies, could improve the model’s ability to learn and distinguish these classes more effectively.

Another limitation of our study is the exclusion of less common sample types, such as gram-negative cocci, slides with mixed bacterial morphologies, and non-bacterial pathogens like yeast. These organisms, including anaerobic GNRs and GPRs, pose unique challenges in Gram stain interpretation due to factors such as over- or under-decolorization, and, less commonly, the presence of spores or rudimentary branching. The absence of sufficient clinical positive cultures of these organisms in our data set limited their inclusion in model training. Additionally, the single-site nature of our data collection further constrained the diversity of included samples. Including these challenging sample types would enhance the utility of GramViT as a tool for laboratory technologists, and represents a critical step toward clinical implementation. To overcome this limitation, we plan to collaborate with multiple institutions to collect a more comprehensive data set that includes these organism types in future studies.

Other limitations include the processing time of each sample. On average, it takes 10 minutes to scan a slide, 1 minute for image preprocessing, and 5 minutes for image analysis, resulting in a total of 16 minutes per sample on the Nvidia RTX A6000 GPU. This workflow could be improved by integrating batch slide scanners that operate overnight, allowing preprocessing and analysis to be performed in batches during off-peak hours. While this approach would reduce wait times and enhance overall efficiency, it would not be compatible with the necessary rapid turnaround time for Gram stains from positive blood cultures or sterile sites such as cerebrospinal fluid. Additionally, strong results on small image crops from the MHU data set suggest that GramViT could effectively analyze images captured with a standard microscope equipped with a camera. Although these images are less comprehensive than WSI analysis, they would enable rapid characterization and be accessible to labs without slide scanners.

Additionally, the lack of large-scale, publicly available Gram stain data sets restricted further exploration of model generalizability. To address these limitations, future work will focus on expanding data collection across multiple sites and phases, as well as closer collaboration with clinical providers. This will facilitate the transition of GramViT from a proof-of-concept model to a clinical laboratory tool. Beyond blood cultures, future studies should also explore the potential application of GramViT to high-volume specimens, such as commonly processed clinical samples, including wound, respiratory, and superficial cultures. This could help streamline workflows in clinical microbiology laboratories, improving efficiency by reducing manual reading time, particularly for high-volume specimens, while complementing the use of rapid molecular diagnostics for certain specimen types. A key aspect of this transition will involve developing a visualization platform that leverages vision transformer attention weights to highlight critical regions of slides for clinical review. Within this context, incorporating GramViT as a pre-screening tool in the clinical workflow would allow laboratory technologists to focus on highlighted regions of interest, facilitating faster and more accurate verification of the model’s analysis.

Given the clinical significance of BSIs and the crucial role of Gram stains, alongside rapid molecular diagnostic assays, in informing early treatment decisions, this work establishes a robust framework for developing a practical tool to assist clinical microbiology laboratories in characterizing Gram-stained bacteria more quickly and reliably. Our findings demonstrate that GramViT is a scalable model capable of handling expanding data sets without the need for time-consuming patch-level annotations, while effectively learning features that generalize across Gram stain images from different laboratories. The potential clinical impact of this work is substantial, as it could streamline diagnostic workflows, reduce diagnostic delays, and enhance the accuracy of early infection management, ultimately improving patient outcomes in critical care settings. By providing a more efficient and reliable approach to bacterial classification, GramViT has the potential to significantly advance the standard of care in microbiology laboratories.

## Data Availability

The code for GramViT is available at: https://github.com/BMIRDS/GramViT.
